# Familial Tuberculosis Mimicking Advanced Ovarian Cancer

**DOI:** 10.1155/2009/736018

**Published:** 2009-12-21

**Authors:** Fakhrolmolouk Yassaee, Farah Farzaneh

**Affiliations:** Infertility and Reproductive Health Research Center, Taleghani Hospital, Medical University of Shahid Beheshti, Tehran 1985717443, Iran

## Abstract

Genital TB may present as on abdominopelvic mass mimicking ovarian malignancy because clinical and laboratory findings are similar. Family history is very important and should be considered for differential diagnosis. Three cases of genital TB with presentation of abdominopelvic masses and with no signs and symptoms of TB were presented. Two of them had positive family history of pulmonary TB. Tissue diagnosis was the best method for diagnosis of genital TB, but it should be reminded that if positive family history of TB was present, mini laparotomy should be done to take biopsy and to make rapid diagnosis before treatment.

## 1. Introduction

Tuberculosis (TB) is still a major cause of morbidity and mortality around the world and the second leading infectious cause of death among adult globally [1]. The risk factors for the development of tuberculosis include immigration, low income populations, immunosuppression, human immunodeficiency virus, and living in close contact with patients suffering from tuberculosis [2, 3]. The precise incidence of genital TB cannot be determined with certainty as some cases are asymptomatic and uncovered accidentally during investigation of infertility. In developing countries, genital TB may account for 3% or more of patients with infertility [1]. The symptoms and signs of abdominopelvic TB can mimic peritoneal carcinosis or ovarian malignancy [4]. Based on Iranian National Health Program, it is more than 50 years that all Iranian newborn will receive vaccination against TB after delivery and before releasing from the hospital. 

But in cities close to the country borders, cases of TB have been reported mainly due to immigration from neighborhood countries particularly Afghanistan, Pakistan and Iraq. Although pulmonary TB can be diagnosed by its particular signs and symptoms as well as laboratory and imaging assessment, peritoneal TB has common symptoms with advanced ovarian carcinoma, including pelvic pain, mass, ascites, and elevated serum CA125 levels. Based on literature review some other case reports showed ambiguity in the differentiation between peritoneal tuberculosis and advanced ovarian cancer before operation [2, 5–7]. Therefore, preoperative diagnosis between these two distinct diseases continues to be a dilemma. We presented three cases of peritoneal tuberculosis mimicking ovarian malignancy to point out the importance of the histopathologic diagnosis before chemotherapy in women suspected having advanced ovarian cancer, especially if there is a family history of pulmonary TB or infertility.

## 2. Case Reports

Three women with abdominopelvic TB were operated during one year in Taleghani Hospital with preoperative suspicious of ovarian malignancy. The demographic and clinical data of these women are summarized in Table 1.

**Case 1**

**Figure 1:**
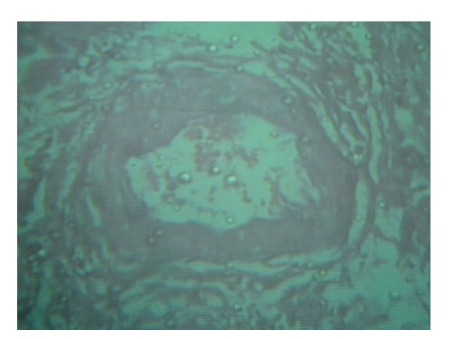
Microscopic picture showing chronic granulomatous inflammation with epitheloid histiocytes and Langhans type multinucleated giant cells (Case 1).

A 24-year-old married, nulligravid woman was referred from gasteroentrology ward to the gynecologic ward with the complain of abdominal pain and distension, nausea, on and off vomiting in the preceding 3 months. Her past medical history was unremarkable but in gynecologic history hypomenorrhea was highlighted (Figure 1). Family history revealed that her father had been diagnosed with pulmory TB, also her cousin had been involved with genital TB. During physical examination, she was a pale young lady with a distended abdomen and a fixed mass palpated up to the umbilicus.

In her paraclinic investigations, among all serum tumor markers related to ovarian cancer, there was only a mild elevation of CA-125 (45 U/mL) Hemoglobin was 9.0 mg/dL. As she had a positive family history for TB a chest X-ray and skin tuberculin test have been performed and both were negative for TB.

Pelvic ultrasonography showed a normal size uterus with endometrial thickness equal to 5 mm. There was a septated multicystic mass of 134 × 140 × 81 mm cube in right adnexa with extension toward the left adnexa. Left ovary was also bigger than normal (70 × 35) mm containing multiple follicles. The ascites in the abdominopelvic cavity with spreading behind the liver was present. Sonographic impression was mucinous cystadenoma of ovary or hydatid cyst.

Abdominopelvic computed tomography showed large amount of ascites in peritoneal cavity with cystic and solid vegetative mass, mild thickening of peritoneal layers in lower abdomen suggesting ovarian mucinous cystadenocarcinoma with peritoneal metastasis was noted. Other possibilities suggested were primary carcinoma of mesenteric, peritoneal like mesothelium, or papillary carcinoma of peritoneum.

Because of highly suggestion of ovarian malignancy preoperation, the patient underwent exploratory laparatomy with midline incision. Total volume of 400cc ascitic fluid was aspirated and sent for cytology and culture. There were severe adhesions and miliary seeding all over the abdominal and pelvic organs. Adhesions were released. Then left ovary 5 × 5 × 6 cm appeared with hemorrhagic cyst and adhesion to the left fallopian tube. So left salpingo oophorectomy was done and the specimen was sent for frozen sections. The result of frozen sections was granulomatous inflammation compatible with tuberculosis. So peritoneal cavity was washed with normal saline and closed. The result of permanent pathology was chronic granulomatous salpingitis compatible with tuberculosis. Ovary: hemorrhagic cyst of corpus luteum. Patient discharged with antituberculosis drugs.

**Case 2**

**Figure 2:**
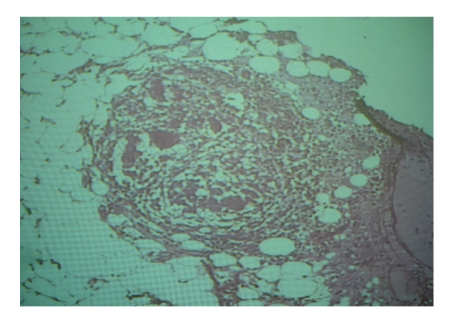
Microscopic picture showing chronic granulomatous inflammation with epitheloid histiocytes and Langhans type multinucleated giant cells (Case 2).

A 39 year–old married woman Gravida 6, para 6 has been transferred to the gynecologic ward from the gasteroentrology ward with the complain of abdominal pain, weight loss and fever in the last 4 months (Figure 2). Her past medical history was unremarkable, and in her surgical history, last 2 deliveries were through cesarean section and she performed the tubal ligation. It is worth to mention that her normal menstrual pattern has been changed to oligomenorrhea since last 6 months. She had also a positive family history; her mother had been diagnosed with pulmonary TB.

Her abdominopelvic examination revealed a fixed and tender mass in the pelvis. As Case 1, due to positive family history for TB, a chest X-ray and skin tuberculin test has been performed and both were negative for TB.

Profiling of serum tumor markers and revealed a high level of CA-125: 207 u/mL. ESR: 27. Hemoglobin was 10 mg%.

Pelvic ultrasonography disclosed uterus and adnexa as a large complex cystic mass shifting to the right with extensive adhesion to the adjacent structures. As the preoperation assessment was in favour of ovarian malignancy, the patient underwent exploratory laparatomy with a midline incision. There was no ascites, but miliary seeding all over the peritoneum and small bowel and large bowel, uterus, fallopian tubes and ovaries were seen, which were biopsied. A confluent mass containing uterus, ovaries and fallopian tube was seen which was necrotic. The frozen sections of the biopsied specimens showed granulation tissue, but as the family was completed and the mass was necrotic, hysterectomy and bilateral salpingo oophorectomy have been done. Peritoneal cavity was washed with normal saline and closed. The permanent result of pathology showed many granulomatous lesion comprised epithelial cells, histiocytes, giant cells. Right ovary and right tube were unremarkable, left ovary, fallopian tube and uterus had granulomatous lesion consistent with tuberculosis. Patient discharged with anti tuberculosis drugs.

**Case 3**

**Figure 3:**
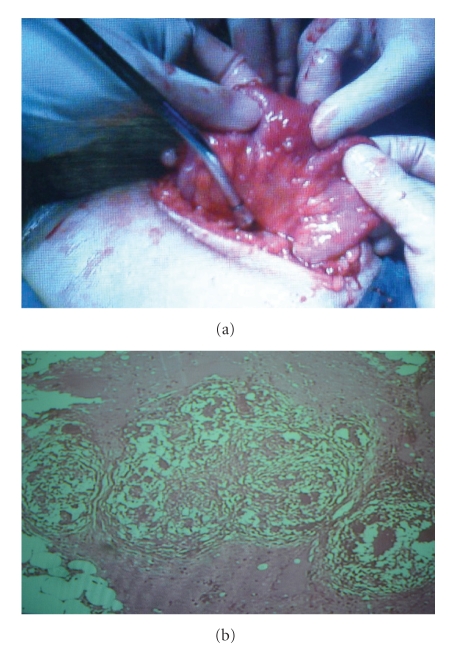
(a) Miliary pattern all over the abdominopelvic organs (Case 3), (b) Microscopic picture showing chronic granulomatous inflammation with epitheloid histiocytes and Langhans type multinucleated giant cells (Case 3).

A 57-year-old married woman, Gravida 9, Para 8, consulted with the gynecologic ward by gasteroentrology consultant and then admitted complaining abdominal pain and distension since last two months (Figure 3). She lost 10 kg during these 2 months which is highly suggestive of a serious problem. Her past medical and gynecologic history was unremarkable. Family history was negative. In physical examination she was pale. Abdomen was distended with tenderness in the palpation of the periumbilical and hypogastric areas. Hemoglobin was 9.4 mg/dL. Serum tumor markers were normal except CA-125 >1000 u/mL. Chest X-ray was normal. Ultrasonography showed normal liver, spleen, kidneys, and uterus, but showed cystic masses in both adnexa with septation in favour of malignancy. Computerized tomography showed ascites. No sign of metastasis. So laparatomy with midline incision was done with impression of ovarian malignancy.

About one liter ascites aspirated and omental cake with miliary seeding, which were seen all over the peritoneum and pelvic organs and over the liver and spleen, has been biopsied. Ovarian masses were removed and sent for frozen sections along with the other biopsied specimens with the result of granulomatosis. Because of necrotic and hemorrhagic appearance of uterus and ovaries as well as the patient's age and condition total abdominal hysterectomy and bilateral salpingooophorectomy were done to prevent postoperation complications. Result of permanent pathology was fibrotic right oviduct with granulomatous inflammation. Right ovary with granulomatous inflammation in favour of TB ascitic fluid cytology showed inflammatory cells. Patient was discharged with antituberculosis drugs.

All three cases were in very good status in 6 months followup with normal pelvic exam, normal pelvic ultrasonography and CA125 measurement.

## 3. Discussion

Peritoneal TB with nonspecific symptoms mimicking ovarian malignancy is a serious problem especially in developing countries. Diagnosis of peritoneal TB before operation is not easy, there is no particular laboratory or imaging assessment to differentiate this disease from advanced ovarian cancer. In our 3 cases diagnosed with abdominopelvic TB, two were in reproductive age (Cases 1 and 2) and one postmenopausal (Case 3). All the cases were admitted firstly to gasteroentrology due to abdominopelvic pain and distension and then transferred to gynecology oncology ward with the diagnosis of advanced ovarian malignancy. Adnexal mass suspicious of malignancy in CT scan and/or sonography and elevated CA125 were seen in all cases. Ascites in Case 1 and Case 3 was detected. Family history for pulmonary TB was positive in Cases 1 and 2 and genital TB for Case 1. The first two cases had a history of oligomenorrhea and hypomenorrhea in the last year before surgery. Due to the positive family history, peritoneal TB was suspected in the first two cases preoperatively. As previous case reports [7, 8] in all our 3 cases non-invasive tests, such as acid-fast stain and culture of the ascitic fluid, ultrasonographic features of the abdomen and pelvis, CT, as well as serum CA125 level were nonspecific for differentiating abdominopelvic TB from ovarian malignancy.

In our 3 cases, because of ambiguity of the results of non invasive tests, explorative laparotomy with a midline incision has been done before treatment. Ascites or peritoneal washings were sent for cytology as well as bacteriologic examination which was negative for both malignant cell as well as TB Miliary deposits all over. The omentum or peritoneal surfaces were seen and assessed by frozen-section analysis, which detected granulamotous inflammation with central necrosis highly suggestive of tuberculous peritonitis. 

Therefore, although tuberculin skin test may be helpful but might be negative, as in our cases, it might be positive because vaccination is a national program in our country.

Chest X-ray may also be normal in women with abdominopelvic TB. The CA125 level, which is elevated in more than half of early and two thirds of advanced epithelial ovarian malignancy [3], can be increased in peritoneal TB [9, 10]. 

Predictive value, specificity, and sensitivity of this marker to reveal epithelial ovarian malignancy are less in premenopausal than postmenopausal due to other benign conditions [11, 12].

Therefore, in areas where TB is endemic, in premenopausal women with elevated CA125, this infection should be considered. 

Some studies assessed new diagnostic tools to differentiate abdominopelvic TB from malignamcy before treatment. 

Ginesu et al. (1998) studied the polymerase chain reaction for mycobacteria but they reported a negative results in cases of peritoneal tuberculosis mimicking advanced ovarian cancer [13].

Tinelli (2008) [8] suggested that abdominopelvic laparoscopy with histopathological findings plus enzyme-linked immuno-spot can confirm the suspect of AP-TB. Although we were unable to perform the last two investigations, their validity is still under investigation. Consequently, we suggest a laparatomy and biopsy of involved area as the gold standard method for definite differentiation between advanced ovarian malignancy and peritoneal TB especially in endemic areas for TB with limitations of using new or expensive technology. 

Although some studies suggest laparoscopic biopsy for histologic diagnosis, we preferred a minilaparatomy to decrease the complications of laparoscopic entrance to the abdomen with extensive adhesions between loop of intestines, abdominal wall, and pelvic organs seen in abdominopelvic TB.

Although this report was not a case control study but it seems that removing the infected pelvic masses from the field of the operation before beginning the medical therapy is beneficial.

 In conclusion, cytology or histopathologic verification of ovarian malignancy before beginning of aggressive therapy including debulking and or adjuvant chemotherapy is mandatory. 

Based on these cases, even in the presence of symptoms and signs highly suggestive of ovarian malignancy, the presence of familial history of pulmonary and/or peritoneal TB along with complaining of oligomenorrhea or hypomenorrhea with low socioeconomic situation are enough markers for the physician to rule out abdominopelvic TB. 

 Women living on the border of our country, close to immigrants campus are mostly at risk to develop the TB. Therefore, taking a precise personal and family history as well as geographic and socioeconomic status of the patients will be helpful. Considering this, we suggest that all families, friends, and people living in the vicinity of women diagnosed with TB should be called through a national health program to be tested for TB and treated accordingly.

## Figures and Tables

**Table 1:** The demographic and clinical characteristics of the cases.

	Case 1	Case 2	Case 3
Age	24	39	57
Gravity	Go	G6	G9
Symptoms	Nausea, vomiting, abdominal distension/pain	Weight loss, fever, abdominal pain	abdominal distension/pain Weight loss >10 kg
Clinical examination	fixed huge abdominopelvic mass	Fixed pelvic mass	Periumblical tenderness, ascites
Past medical history	Anemia hypomenorrhea	Anemia, oligomenorrhea	Anemia
Familial history	Father: pulmonary TB Cousin: genital TB	Mother: pulmonary TB	Negative for TB
Tuberculin test	Negative	Negative	Negative
Ascites	Yes	No	Yes
CA125 (IU/mL)	45	207	>1000
Chest X ray	Normal	Normal	Normal
Computed tomography	large amount of ascites, thickening peritoneal layers cystic/solid mass	Not performed	Ascites
Ultra-sonography	Septated multicystic mass, ascites	Large complex cystic mass	Bilateral adnexal cystic mass with septation
Preoperative suspicion of TB	No	No	No
